# Cost-effective and eco-friendly copper recovery from waste printed circuit boards using organic chemical leaching

**DOI:** 10.1016/j.heliyon.2023.e13806

**Published:** 2023-02-17

**Authors:** Navashree Nagarajan, Parthasarathy Panchatcharam

**Affiliations:** Department of Electronics and Communication Engineering, CMR Institute of Technology, Bengaluru, 560037, India

**Keywords:** Waste management, Waste printed circuit boards, Chemical leaching, Metal recovery

## Abstract

Electronic waste generation is indeed a global concern; therefore, appropriate management and recycling are becoming highly significant. Printed circuit boards (PCBs) are significant portion of e-waste; contains a large number of valuable metals, rendering this material an important recovery resource. Among all other metals, the high Copper concentration of PCB residues, which is often ten times higher than that of rich-content rocks, makes these residues an appealing secondary source of Cu recovery. The primary goal of this study is to develop a simple and economical method for recovering Cu from waste PCBs. To leach metals, a combination of citric acid, acetic acid, and hydrogen peroxide (H_2_O_2_) was utilized. The influence of systemic factors such as citric acid concentration, acetic acid concentration, and H_2_O_2_ concentration on Cu leaching process was investigated. The results proved that the combination of citric acid, acetic acid, and H_2_O_2_ has increased the leaching efficiency of copper. More copper was dissolved when leaching with 0.5–1.5 M citric acid, 2.5–7.5%, and 2.5–7.5% H_2_O_2_ at 30 °C; however the individual acids produces less amount of Cu such as 26.86 ppm, 22.33 ppm, and 6.28 ppm whereas, high amount of Cu is obtained from the leaching solution containing 1 M citric acid, 5% acetic acid and 5% H_2_O_2_ with 325.89 ppm respectively. Thus, the combination of these acids and can be used as standardized method for leaching of Cu. These findings suggest that organic acids can replace inorganic acids as eco-friendly lixiviants for waste management.

## Graphical abstract

**Figure:**
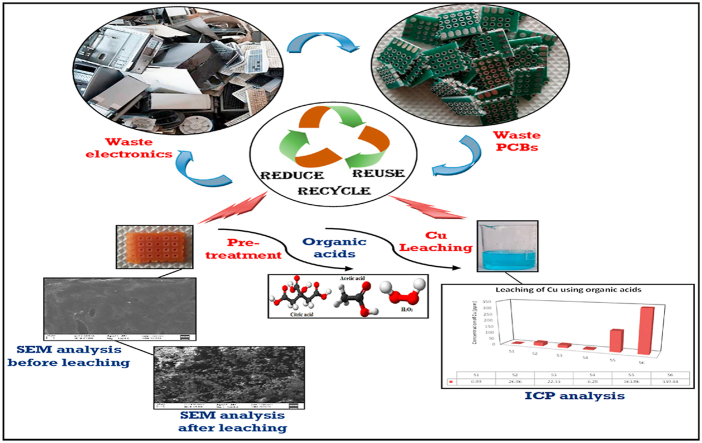

## 1 Introduction

**Fig. .1:**
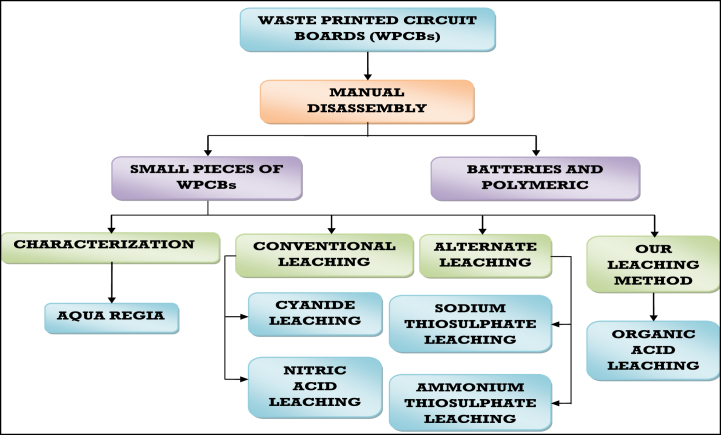
Schematic flow of different types of hydrometallurgical recovery methods.

Electrical and Electronic Equipment (EEE) consumption is a major key to increase the global economic development. The modern society and living standards of the people now-a-days makes the use of electronics indispensable in day to day life. On an average the consumption of EEE increases about 2.5 million metric tons (Mt) globally. Electronic waste (E-waste) is now one of the fastest-growing solid waste categories due to its short lifespan. Approximately 53.6 Mt of electronic waste was generated per capita in 2019. It is estimated that e-waste may exceed to 74 Mt by the year 2030. Thus, as global level e-waste is increasing rapidly with 2 Mt per year. However only 17.4% of the e-waste is only recycled annually [1]. The majority of E-waste contains valuable, hazardous metals such as Al, Ag, Be, Au, As, Bi, Cr, Cd, Cu, Ni, Hg, Pt, Fe, Sb, Zn, and Si, as well as organic chemicals and flame retardants [2]. E-waste is hazardous to both the environment and human health owing to the presence of toxic components and multi-metals. Heavy metals, organic and inorganic components from e-waste affects water resources when they leach into bodies of water [3]. Previously, available ores possessed 5.0% or more Cu content, while grades of most ores at present contain less than 1.0% metal content. When the metal content of E-waste is evaluated, it is called an urban mine, and if correctly processed, it has the potential to be an alternative source of resource recovery [[4], [5], [6]]. E-waste is either improperly dumped in landfills or burned in open air. Dioxins and furans are produced, as well as hazardous waste residues runoff and pollute the soil, water, and air. Metal leaching from e-waste is critical not only for waste management sustainability, but also for satisfying an increasing demand for these metals while conserving rapidly depleting natural resources [7,8]. Industrial treatment systems for metal leaching from E-waste use pyrometallurgical and hydrometallurgical techniques. There are some drawbacks to pyrometallurgical techniques. The pyrometallurgical technique can only achieve a partial separation of metal, which is primarily designed for base metals and inefficient for precious metal leaching [9]. Hydrometallurgical techniques have received a lot of interest recently for the treatment of E-waste. Strong acids are effective as metal-leaching agents for numerous researchers. However, the environment is endangered by the discharge of SO_3_, NO_x_, Cl_2_, and acidic wastewater during the leaching. Therefore, the development of eco-friendly hydrometallurgical technologies is required for use in the future. A good substitute is the use of organic acids, particularly citric acid, and acetic acid. Several studies have employed citric acid and H_2_O_2_ to leach metals from waste lithium cobalt batteries, sewage sludge, and scrap battery paste [10]. Various methods of hydrometallurgical metal recovery are illustrated in Fig. 1.

In water, citric acid dissolves effortlessly. Both aerobic and anaerobic environments cause it to deteriorate. As a result, it is simple to treat the waste solutions produced following metal leaching. Researchers employed citric acid and H_2_O_2_ to extract metals from used lithium-ion batteries. With a high reduction potential of 1.78 V, H_2_O_2_ is a powerful oxidant [11]. It was previously widely employed as an oxidant in acid SO_4_ solutions to leach metals from old PCB powder [9]. Low molecular weight organic acids like acetic acid are frequently referred to as moderately and weakly chelating agents. It has been widely employed in a variety of industries, including manufacturing in the medical field, and the food industry. Acetic acid was discovered to be more effective for recovering metal from lead-free and tin-copper solders [12]. For the leaching of metals from used PCB, the combination of citric acid, acetic acid, and H_2_O_2_ has not yet been tested. The current endeavor is an effort to create a hydrometallurgical process that leaches metal from waste PCB using H_2_O_2_ and environmentally acceptable acids. The amount of metals leached depends on how well organic acids can couple with metal ions. Thus, metal leaching is greatly facilitated by the production of a more stable ligand.

## 2 Materials and methods

Acetic acid, citric acid, hydrogen peroxide, and deionized water purchased are of analytical grade. The waste printed circuit boards (WPCBs) were gathered from the scrap market. RAM, chip slots, and PCI slots that were attached to PCBs were physically removed, and they were then sliced into 3 × 3 cm pieces. To remove any dirt, oil, chemicals, adhesives, smears, etc., the WPCB samples were then washed and rinsed with distilled water and acetone. They were allowed to dry for 24 h at room temperature.

### 2.1 Chemical coating removal from WPCBs

WPCBs have a layer of coating on its surface that prevents the metal leaching agent from passing through it and leaching through the WPCBs. The sodium hydroxide (NaOH) treatment has been used in the current investigation to remove the chemical layer on WPCBs [13]. After being soaked in a 5 M NaOH solution for the entire night, the WPCBs were rinsed under running water until the adhering NaOH was eliminated. By measuring the pH of the cleansed water, this was observed. The pH of the cleaned water is neutral, which confirms the total elimination of NaOH. The cleaned WPCBs were then utilized for further investigations. Following NaOH treatment, the metal concentration was examined from the sample.

### 2.2 Leaching procedure

**Fig. 2:**
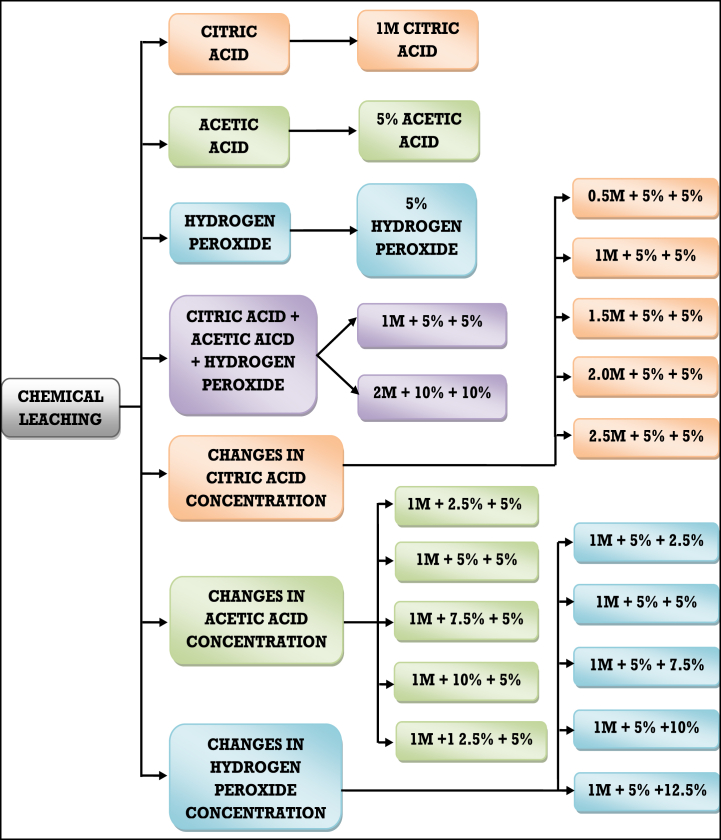
Chemical leaching using various concentrations of citric acid, acetic acid and hydrogen peroxide.

The WPCB pieces (3 × 3 cm) were submerged in a solution of citric acid, acetic acid, and H_2_O_2_ in 100-ml beakers for all of the metal leaching studies. To examine the metal leaching from WPCB, five control tests were conducted. The leaching solution for the first control experiment contains citric acid and 50 ml of deionized distilled water. The leaching solution for the second control experiment comprised 50 ml of the acetic acid solution, while the third control contained 50 ml of H_2_O_2_ solution. A 50 ml mixture of citric acid, acetic acid, and H_2_O_2_ was used as the fourth leaching control, and a 50 ml mixture of an enhanced concentration of these chemicals was used as the fifth control. The workflow parameters were evaluated as described in the following sections and also listed in Fig. 2 given below. Various concentrations (0.5–2.5 M) of the 25 ml citric acid solutions were prepared separately for this experiment in various beakers. The 5% acetic acid (25 ml) and 5% H_2_O_2_ (50 ml) were produced and added to the citric acid solution. Whereas, impact of acetic acid concentration was investigated using 25 ml solutions in a range of concentrations (2.5–12.5%). Then, to the acetic acid solution, 5% H_2_O_2_ (50 ml) and 1 M (25 ml) citric acid were added. Similarly, to study the impact of H_2_O_2_ concentration, 50 ml of H_2_O_2_ (30% w/v) solutions in a range of concentrations (2.5–12.5%) were used. The H_2_O_2_ solution was then mixed with 5% acetic acid (25 ml) and 1 M citric acid (25 ml). Then, each of these beakers was added with 2.5 g of WPCB pieces. The beakers were incubated in an incubator for 24 h in a static environment without stirring at ambient temperature.

## 3 Result and discussion

In the current investigation, experiments were done to see how metals might leach from WPCB pieces. As lixiviants, acetic acid and citric acid were utilized. H_2_O_2_ was added to the organic acids to speed up the metal leaching. The potential leaching mechanism as well as its impact on concentration was examined below.

### 3.1 Reaction mechanism of metals with leaching agents

Several mechanisms can be used to explain how metals dissolve in e-waste. It is possible to explain the system's potential mechanism using simply H_2_O_2_. In its interactions with metals, H_2_O_2_ can take the role of either an oxidant or a reductant [14]. A generalized mechanism for H_2_O_2_-induced metal oxidation can be provided by the following reaction (1) and (2)

(1) $$M+H_{2}O_{2}\to \;M^{2+}+OH+OH$$

(2) M+OH→M2++OH

Where, M stands for metal.

For the proton-promoted dissolving process, organic acid disintegrates to produce H+. Metals can be dissolved by organic acids by providing protons and ligands. By forming complexes and acidifying the metallic components in E-waste, they can dissolve them which are represented in equations (3), (4), (5).

(3) $$RCOOH+H_{2}O\;↔\;{RCOO}^{-}+H_{3}O+$$

Proton reduction produces hydrogen and oxidizes the metal,

(4) $$2H_{3}O^{+}+2e^{-}\to \;H2+2H2O$$

(5) M→M2++2e

The ligands in organic acids, like citrate (Cit) from citric acid, generate stable metal complexes in a complexation mechanism. Metals may dissolve more readily in solutions as a result of the complexation reaction which is represented in equations (6), (7), (8) [[15], [16], [17], [18]]:

(6) $${RCOO}^{-}+M\;\left(H_{3}O^{+}\right)\;\to \;RCOOM+H_{2}O$$

The findings of the current investigation demonstrate metal leaching in 24 h by employingH_2_O_2_, citric acid, or acetic acid. The production of peroxy carboxylic acid may be the cause of the increased ability of organic acid to leach metal in the presence of H_2_O_2_

(7) $$R-COOH+H_{2}O_{2}\to R-COOOH+H_{2}O$$

The metal can be easily oxidized by strong oxidants known as peroxy carboxylic acids. In an aqueous medium, its instability causes an impulsive breakdown in which each peroxy carboxyl group (-COOOH) consumes two electrons, similar to H_2_O_2_ [19].

(8) $$R-COOOH+M+2H\to R-COOH+H_{2}O+M^{2+}$$

In another possible mechanism, metals from e-waste can form metal hydrogen citrate/acetate in the presence of H_2_O_2_, citric acid, and acetic acid via the reaction described below in equation (9) and also represented in Fig. 5.

(9) $$C_{6}H_{8}O_{7}/\;C_{2}H_{4}O_{2}+M+H_{2}O_{2}\to \;M\;\left(C_{6}H_{8}O_{7}\right)\;/\;M\;\left(C_{2}H_{4}O_{2}\right)+H_{2}O+M^{2+}$$

(Citrate) (Acetate)

### 3.2 Kinetic models

Though the reaction mechanism can be predicted, it can also be confirmed by using the various kinetic models.

#### 3.2.1 Diffusion-controlled kinetic model

The solid-state reaction can be expressed in the form of kinetic equation as follows (10–16):

(10) $$\frac{dx}{dt}=r.f\left(x\right)$$

Where, *r* is the reaction rate constant, *t* is the time and *x* is the reacted fraction:

(11) $$x=\frac{m_{1}-m_{t}}{m_{1}-m_{2}}$$

Where, *m*_*1*_ is the initial mass*, m*_*2*_ is the final mass, and *m*_*t*_ is the mass at any time. Therefore the integration equation represented as:

(12) $$g\left(x\right)=\int_{0}^{x}\frac{dx}{f\left(x\right)}=r\int_{0}^{t}dt=r.t$$

There are several other diffusion-controlled models that are suitable for metal leaching process. They are as follows:

(13) $${\left[1-{\left(1-x\right)}^{\frac{1}{2}}\right]}^{2}=r.t$$

(14) $${\left[1-{\left(1-x\right)}^{\frac{1}{3}}\right]}^{2}=r.t$$

(15) $${\left[{\left(1-x\right)}^{\frac{-1}{3}}-1\right]}^{2}=r.t$$

(16) $${1-{\frac{2x}{3}-\left(1-x\right)}^{\frac{2}{3}}]}^{2}=r.t$$

The g(*x*) plotted against time and rate of the reaction gives the straight line and its slope can be obtained. These co-efficient leads to the determination of linear regression (R^2^). Thus, these models help to find out the appropriate kinetic model for metal leaching from waste PCBs.

#### 3.2.2 Pseudo-reaction-order kinetic models

The other kinetic model apart from diffusion-controlled models is pseudo-reaction-order kinetic models. This can be expressed as in equation (17):

(17) $$\frac{\partial y_{t}}{\partial t}=r_{1}\left(y_{e}-y_{t}\right)$$

where, $y_{t}$ is the rate of metal recovery at any time, $r_{1}$ is the rate constant of the pseudo first-order reaction and $y_{e}$ is the rate of metal recovery at equilibrium. With the pseudo-first order rate constant, straight line can be obtained along with the slope on the linear regression line which helps to determine the maximum metal recovery rate. Similarly, this can also be analyzed by using pseudo-second- order kinetic model which can be expressed as in equation (18):

(18) $$\frac{\partial y_{t}}{\partial t}=r_{2}{\left(y_{e}-y_{t}\right)}^{2}$$

Where, $r_{2}$ is the rate constant of the pseudo first-order reaction.

With the help of this kinetic model, linear regression (R^2^) values are estimated and correlation between the metals on acid medium can be determined and compared as discussed by the previous researchers in their studies [20].

### 3.3 SEM and ICP analysis

**Fig. 3:**
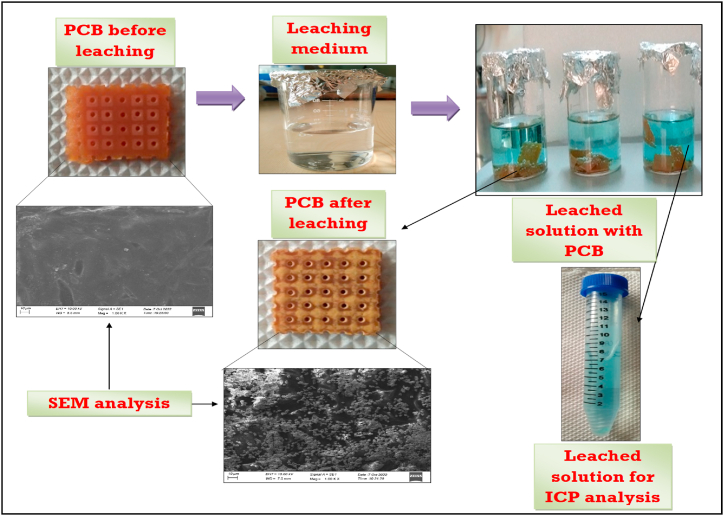
WPCBs before and after leaching.

**Fig. 4:**
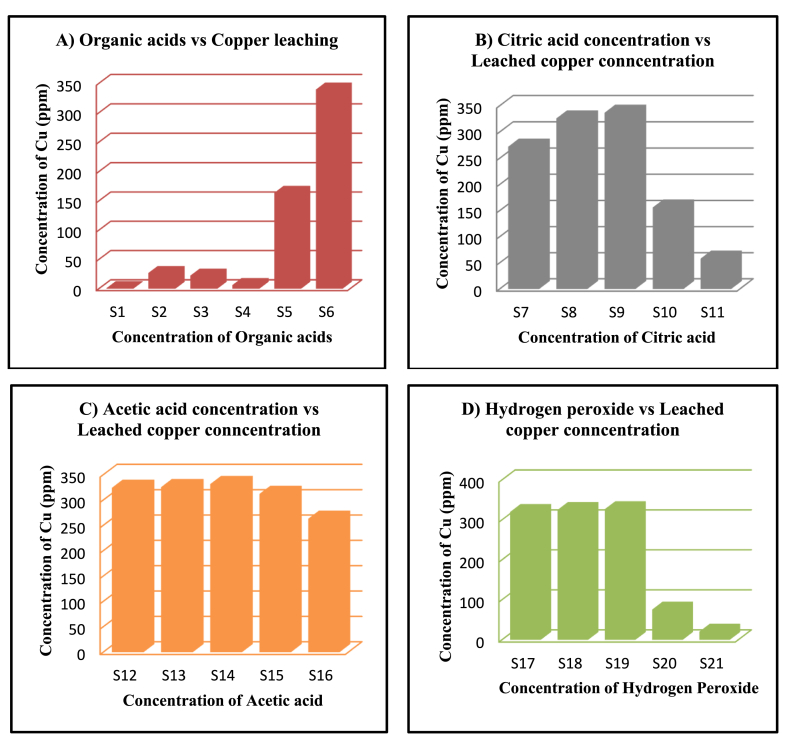
A) ICP analysis of copper leaching- S1 (NaOH), S2 (Citric acid), S3 (Acetic acid), S4 (H_2_O_2_), S5 (Citric acid + Acetic acid + H_2_O_2_), S6 (increased concentration of Citric acid + Acetic acid + H_2_O_2_), B) ICP analysis of copper leaching under varying citric acid concentration – S7 (0.5 M), S8 (1.0 M), S9 (1.5 M), S10 (2.0 M), S11 (2.5 M), C) ICP analysis of copper leaching under varying acetic acid concentration – S12 (2.5%), S13 (5%), S14 (7.5%), S15 (10%), S16 (12.5%), D) ICP analysis of copper leaching under varying H_2_O_2_ concentration – S17 (2.5%), S18 (5%), S19 (7.5%), S20 (10%), S21 (12.5%).

During the Cu leaching experiments, samples were collected at regular intervals and sent for ICP-OES (Inductively Coupled Plasma - Optical Emission Spectrometry) analysis to assess the Cu content. According to the ICP-OES analysis, NaOH treatment results in the release of 0.89 ppm of Cu ions, emphasizing the loss of a trace amount of Cu metal ions from the WPCBs. The NaOH treatment on WPCBs is represented by S1 in Fig. 4(A). Scanning Electron Microscope (SEM) testing with the following SEM instrument model: EVO 18 was used to examine the change in surface morphology of WPCBs before and after leaching. Fig. 3 depicts a SEM analysis of the WPCB piece before and after leaching. Before leaching, the WPCB piece had a layer of copper on its surface. The WPCB was then treated with citric acid, acetic acid, and H_2_O_2_ in varying concentrations. The rough surface of the WPCB piece is visible after the leaching process, confirming the Cu metal leaching from the WPCB piece from SEM analysis.

**Fig. 5:**
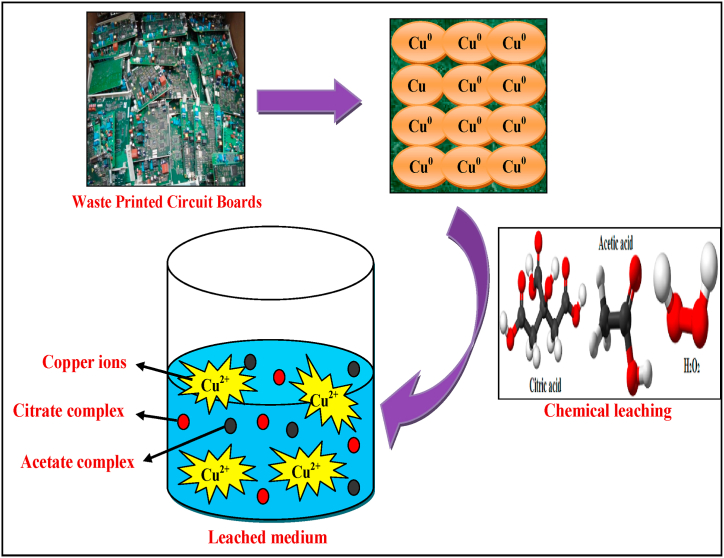
Mechanism of organic chemical leaching of Copper ions from WPCBs.

The WPCBs treated with citric acid, acetic acid, and H_2_O_2_ as control were subjected to ICP analysis which shows the individual treatment of citric acid, acetic acid and H_2_O_2_ leaches 26.86 ppm, 22.33 ppm, and 6.28 ppm. Whereas the combination of these three with 1 M concentration of citric acid, 5% of acetic acid, and % of H_2_O_2_ shows 163.86 ppm and the enhanced concentration of this combination shows 339.44 ppm of copper leaching. Thus, these results indicate that the individual acids have less efficiency of leaching while the combination of these acids leaches a significant amount of copper in the solution. Fig. 4(A) represents the concentration of copper ions leached from the treatment of the various organic acids and their combinations.

### 3.4 Influence of citric acid concentration

On metal leaching, the impact of citric acid concentration was examined. The metal leaching procedure was carried out for 24 h at 30 °C under static circumstances using 5% H_2_O_2_ and 5% acetic acid, and the citric acid concentration ranged from 0.5 to 2.5 M. The results of ICP analysis show that the leaching of Cu is 272.11 ppm, 325.98 ppm, 336.81 ppm, 156.21 ppm, and 58 ppm at various concentrations such as 0.5, 1.0, 1.5, 2.0, and 2.5. Fig. 4(B) depicts the results. According to these findings, increasing the concentration of citric acid increases the leaching mechanism up to 1.5 M, and increasing the concentration of citric acid further decreases the leaching mechanism. As a result, the concentration of citric acid influences metal leaching. According to previous research, the leaching efficiency decreases with increasing organic acid concentration [11,21]. In contrast to these findings, another study found that increasing the concentration of sulphuric acid did not enhance leaching of Cu and Zn [22]. According to the findings of this study, the optimum concentration of citric acid for copper leaching is 1.5 M. When the concentration of citric acid is increased to 1.5 M, the leaching behavior improves. This is due to the formation of metal complexes with citrate, which are eventually degraded by H_2_O_2_ to release free metal ions in the solution. As the concentration rises further, the acid forms more complexes with the metals but is unable to convert them to metal ions due to a lack of H_2_O_2_, resulting in a decrease in leaching efficiency.

### 3.5 Influence of acetic acid concentration

Metal leaching was investigated in relation to acetic acid concentration. Acetic acid concentrations ranged from 2.5% to 12.5% and metal leaching process was carried out for 24 h at 30 °C under static conditions with 5% H_2_O_2_ and 1 M citric acid. The leaching of Cu at various concentrations such as 2.5, 5.0, 7.5, 10, and 12.5% is 324.24 ppm, 325.6 ppm, 331.69 ppm, 312.3 ppm, and 263.27 ppm, according to the results of ICP analysis. The results are shown in Fig. 4(C). According to these findings, increasing the concentration of acetic acid increases the leaching mechanism by up to 7.5%. Increasing concentration of acetic acid results in slight decrease in the leaching capacity. Thus, acetic acid concentration has a moderate effect on metal leaching. These findings are also consistent with the findings of previous studies conducted by the researchers [11,21]. In contrast to these findings, another study discovered that increasing sulphuric acid concentration did not improve Cu and Zn leaching [22]. According to the findings of this study, the optimum concentration of acetic acid used for copper leaching is 7.5%. The increase and slight decrease in leaching are due to the same mechanism used by citric acid. Fig. 7 represents the leaching mechanism of copper ions using the organic acids.

### 3.6 Influence of hydrogen peroxide concentration

The amount of H_2_O_2_ used, the concentrations of citric acid and acetic acid, and the leaching temperature are just a few of the variables that affect the metal leaching process. To optimize these characteristics, experiments were conducted. Using a 1 M citric acid solution and 5% acetic acid solution with different H_2_O_2_ concentrations (2.5%–12.5%), the impact of H_2_O_2_ concentration on metal leaching from WPCB pieces was examined at 30 °C for 24 h. Fig. 4(D) illustrates the findings. The amount of Cu leached at 2.5, 5.0, 7.5, 10, and 12.5% concentrations are 320.16 ppm, 325.23 ppm, 327.11 ppm, 75.96 ppm, and 20.85 ppm, as per the results of ICP analysis. Metal leaching increased significantly with increasing H_2_O_2_ concentration up to 7.5%, while increasing H_2_O_2_ concentration further decreases metal leaching efficiency. As a result of this research, the leaching efficiency was determined to be optimal at 7.5%. These findings support those of the researchers earlier study, which had similar findings [11]. After a certain point, an increase in H_2_O_2_ causes more hydrogen ions to form as a result of dissociation, which ultimately reduces the organic acid's ability to dissolve the metals and lowers its ability to leach. Thus, the concentration of H_2_O_2_ in the solution is confirmed to be dependent on copper ion leaching. Previous research on the chemical leaching of waste printed circuit boards using citric acid and H_2_O_2_ found no significant difference in metal ion leaching due to temperature and shaking speed changes [10]. Previous research on the chemical leaching of waste printed circuit boards using citric acid and H_2_O_2_ found no significant difference in metal ion leaching due to temperature and shaking speed changes [12].

### 3.7 Other metal ions leached with copper

Organic acids leach the metal ions potentially from waste printed circuit boards. The acid solutions not only leach copper ions but also other metal ions such as aluminium (Al), iron (Fe), potassium (K), manganese (Mn), nickel (Ni), phosphorus (P), lead (Pb) and zinc (Zn). The concentration of the leached metal ions was too low when compared with the concentration of copper.

**Fig. 6:**
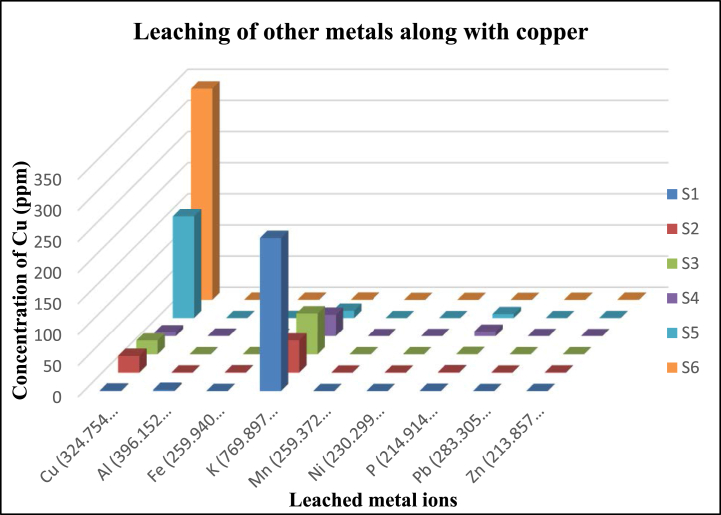
Graphical representation of leached metal ions at different leaching medium: S1 (NaOH), S2 (Citric acid), S3 (Acetic acid), S4 (H_2_O_2_), S5 (Citric acid + Acetic acid + H_2_O_2_), S6 (increased concentration of Citric acid + Acetic acid + H_2_O_2_).

In this case, the NaOH treatment leaches most of the K^+^ ions into the leachant. Treatment of acetic acid, citric acid and hydrogen peroxide also leaches K^+^ ions in moderate amount when compared to Copper. However, the combination of citrate, acetate and H_2_O_2_ leaches copper ions efficiently at higher concentration than other metal ions. Thus, makes it easy to separate other metal ions from the copper ions as they were in very minute quantities and does not interfere further investigation of the copper ions in the solution and proves to be a most efficient method among other methods. The concentration of the metal ions leached with different acid treatment was listed in Table .1 and as graphical representation in Fig. 6.

**Table 1:** Different metal ions leached on organic chemical leaching- S1 (NaOH), S2 (Citric acid), S3 (Acetic acid), S4 (H_2_O_2_), S5 (Citric acid + Acetic acid + H_2_O_2_), S6 (increased concentration of Citric acid + Acetic acid + H_2_O_2_).

SAMPLE	Cu (324.754 nm) (ppm)	Al (396.152 nm) (ppm)	Fe (259.940 nm) (ppm)	K (769.897 nm) (ppm)	Mn (259.372 nm) (ppm)	Ni (230.299 nm) (ppm)	P (214.914 nm) (ppm)	Pb (283.305 nm) (ppm)	Zn (213.857 nm) (ppm)
S1	0.89	2.06	0.12	246.47	0.02	0.03	0.47	0.04	0.14
S2	26.86	0.12	0.43	51.69	0.01	0.01	0.64	0.03	0.13
S3	22.33	0.09	0.22	66.28	0.01	0.01	0.49	0.05	0.08
S4	6.28	0.73	0.15	34.83	0	0.04	7.07	0.03	0.02
S5	163.86	0.2	0.31	11.92	0.01	0.02	6.44	0.03	0.16
S6	339.44	0.48	0.52	0.58	0.01	0.06	0.01	0.13	0.54

**Fig. 7:**
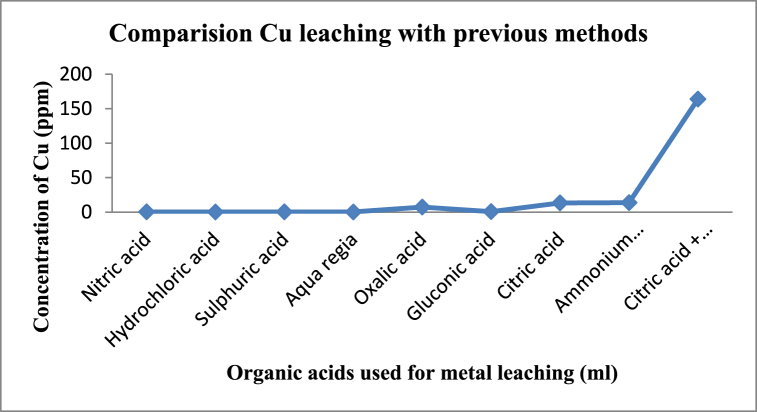
Concentration copper ions leached using various chemical leaching agents.

### 3.8 Comparison of copper leaching with previous methods

Numerous studies have been done for metal recovery from waste printed circuit boards. The acids used for chemical leaching plays vital role in the recovery of metals. Thus, recovery of particular metal ions from the waste PCBs are crucial. However, by analysing the capacity of the different acids for leaching paves the way to determine the efficient acid or combination of acids to retrieve the particular metal ions of our interest. Thus, from the previous literature each individual acid (especially inorganic acids) on leaching provides less amount of copper leaching whereas the leaching using organic acids recover moderate amount of Cu^2+^ ions into the leaching medium. However the combination of these acids (our study) leached copper ions efficiently at higher concentration than other methods in the literature [10,12,23] which was represented in Fig. 7.

Although numerous researchers have studied the leaching of metal ions using acids, our goal is to recover metal ions with less toxicity and at a lower cost. In accordance to this, the combination of citric acid, acetic acid, and H_2_O_2_ has proved to have a higher copper leaching efficiency than the previous studies, which uses only citric acid and H_2_O_2_. Thus, when acetic acid is added to citric acid and H_2_O_2,_ it increases the leaching efficiency when compared to citric acid alone. From this study, it is confirmed that the addition of acetic acid helps to improve the Cu leaching efficiency along with the other acids.

## 4 Application and future perspectives of recovered copper ions

Copper ions recovered by the above leaching methods can be used for various special functions in metal alloy design and the biomedical field.

### 4.1 Role in metal alloy design

**Fig. 8:**
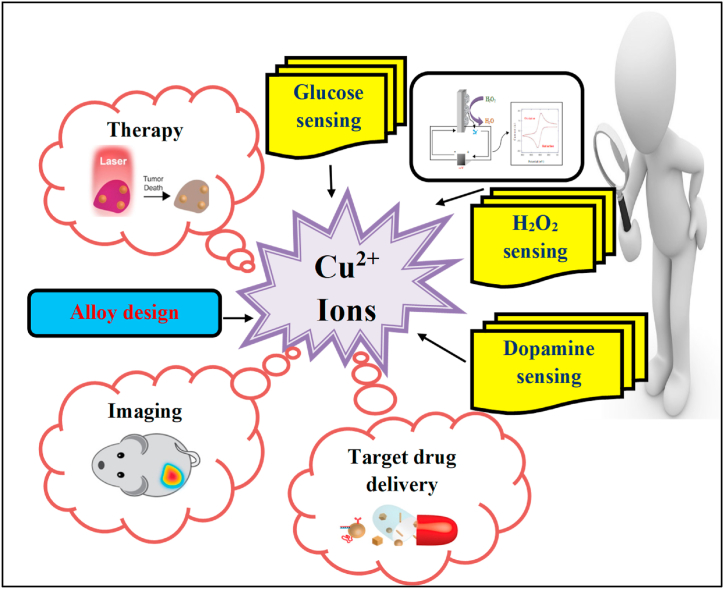
Application of recovered copper ions.

The alloying components of stainless steel are primarily separated into two categories, one is ferrite-forming elements and the other is to encourage the development of elements found in the austenite phase. Copper is responsible for the enlargement of austenite phase, which combine with iron to form an alloy and helps to refine the stainless steel grain size [24]. There are three sorts of alloying elements in a titanium alloy system: a phase, b-phase stabilizing element, and [25] components that have negligible influence on the phase transition temperature. Here, copper acts as b-phase stabilizing element and produce a small amount of alloy solution and lower the phase transition temperature. With an increase in addition of copper, the melting temperature of titanium falls [26] making the casting process easier. Small dental models made of [27]. Ti–Cu alloys can be utilized for dental casting. Ti–Cu alloys exhibited good cast precision and machinability [28]. As a result, recycled copper can be utilized to create metal alloys. Application of copper ions was illustrated in Fig. 8.

#### 4.1.1 Role in biomedical field

The copper that has been recovered from organic chemical leaching can be used to create metal alloys that are frequently employed in the biomedical industry. The discharge of copper ions from the copper alloy Cu-SS was safe for human health and has good biocompatibility [29]. Tumor necrosis factor (TNF)-a expression was also shown to be lower in the bone tissue around 317 L SS than it was in 317 L SS, suggesting that Cu may be able to inhibit the generation of inflammatory factors and so avoid the formation of inflammatory reactions. Cu-SS has also showed its capacity to promote osteogenesis concurrently. Cu-SS alloy inhibits the growth of vascular smooth muscle cells (VSMC), which helps to progress re-endothelialization, and stimulates the proliferation of vascular endothelial cells (VEC), which helps to reduce the development of thrombosis [30]. Compared to conventional NiTi alloys, 316 L-Cu-SS had dual functionalities that were antiencrustation and anti-infection, and it could prevent the growth of biofilm and diminish urease production, lowering the pH of infected urine [31]. Researchers discovered that the Ti–Cu alloy exhibited the best osteoblast adhesion, cytoskeleton development, and cell movement. Cu-containing magnesium alloy also has good biocompatibility and the ability to cure bone injury [32]. The recovered copper ions can be converted to other forms such as nanoparticles and can be used for the development of biosensors for the detection of biomolecules.

## 5 Conclusion

Copper metal is abundant in WPCBs. The purity and quantity of metal in waste PCBs are higher than in rich-content mineral ores. As a result, recycling discarded PCBs benefits both resource recovery and environmental conservation. The current study found that combining organic acids and H_2_O_2_ was just as effective as employing inorganic acids. It was also substantially more successful than the bioleaching technique. It was discovered that the presence of citric acid, acetic acid, and H_2_O_2_is required for copper metal leaching. A 3 × 3 cm WPCB piece was completely leached in 24 h at 30 °C using various concentrations of citric acid, acetic acid, and H_2_O_2_. The usage of small pieces of WPCBs simplified the leaching procedure and proved to have a high leaching efficiency. It solves several of the disadvantages of utilizing powdered WPCBs for metal leaching. Furthermore, it aided in the recycling and reuse of WPCBs for potential copper leaching.

## Author contribution statement

Navashree Nagarajan, Parthasarathy Panchatcharam: Conceived and designed the experiments; Performed the experiments; Analyzed and interpreted the data; Contributed reagents, materials, analysis tools or data; Wrote the paper.

## Funding statement

This work is supported by DST-SERB, New Delhi (EEQ/2021/000467EEQ).

## Data availability statement

Data will be made available on request.

## Declaration of interest's statement

The authors declare no competing interests.
